# Reply: ^99m^Tc-labelled Stealth liposomal doxorubicin (Caelyx®) in glioblastomas and metastatic brain tumours

**DOI:** 10.1038/sj.bjc.6600094

**Published:** 2002-02-12

**Authors:** M I Koukourakis

**Affiliations:** Tumour and Angiogenesis Research Group, Department of Radiotherapy and Oncology, Democritus University of Thrace, PO Box 12, Alexandroupolis 68100, Greece

## Abstract

*British Journal of Cancer* (2002) **86**, 660–661. DOI: 10.1038/sj/bjc/6600094 www.bjcancer.com

© 2002 Cancer Research UK

## Sir

In these previous studies we provided a simple method to label Caelyx by incubation of 5 mg of the ready-to-use solution with 20 mCi of ^99m^Tc-DTPA. Instant thin layer chromatography (ITLC) suggested an 80% labelling (Koukourakis *et al*, 1999), which is also a result found by Dr Laverman and colleagues. As additional more sophisticated analysis, performed by the later research group, failed to confirm this finding, it was suggested that the tumour and body imaging obtained in our studies is rather a result of ^99m^Tc-DTPA and not of labelled liposomes.

^99m^Tc-DTPA is currently used in the evaluation of renal function, and as well noted by Dr Laverman, this is rapidly cleared from the kidneys. Two hours following injection, the imaging quality of kidneys is really poor. ^99m^Tc-DTPA can give good images of gliomas, probably as a result of the high tumour vascularization or even of the disrupted blood–brain barrier, which allows a net contrast between normal and abnormal brain. However, imaging of other tumours with ^99m^Tc-DTPA is questionable.

**Figure 1:**
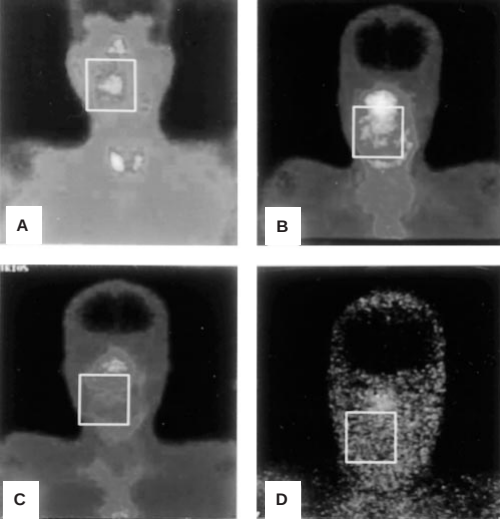
Comparison of images obtained from a patient with a large naso/parapharyngeal tumoural mass (white box marked on figures) using different radio-tracers (injection of 20 mCi, 20 min capture time): (**A**) ^99m^Tc-sestamibi imaging at 2 h post-injection, (**B**) ^99m^Tc-DTPA-Caelyx at 2 h post-injection, (**C**) ^99m^Tc-DTPA at 1 h post-injection and (**D**) ^99m^Tc-DTPA at 2 h post-injection.

We believe that the best answer to whether our simple labelling technique labels Caelyx indeed, comes from the clinical practice. The patterns and quality of imaging using ^99m^Tc-DTPA-Caelyx has been assessed in more than 30 non-small cell lung cancer patients comparatively with ^99m^Tc-sestamibi (Koukourakis *et al*, 1997), while in five of them imaging with simple ^99m^Tc-DTPA was also performed at 2 h post-injection. The patterns of normal tissue imaging using the three radio-pharmaceuticals was entirely different. The quality of tumour images obtained with sestamibi and labelled-Caelyx was very good, while in some cases liposomal imaging was even better. Tumour imaging with ^99m^Tc-DTPA was of unacceptably poor quality, and rather absent in 3 out of 5 cases. Similar comparison of the three imaging procedures in patients with head and neck cancer showed an excellent imaging using labelled-Caelyx and ^99m^Tc-sestamibi, while using ^99m^Tc-DTPA the imaging was questionable especially at 2 h post-injection ( Figure 1

). The superiority of liposomal imaging over sestamibi was confirmed in more than 10 glioma patients. Indeed, lesions of some mm in dimensions were perfectly scanned using ^99m^Tc-DTPA-Caelyx (Koukourakis *et al*, 2000a), which is rarely seen with sestamibi.

We, therefore, have to cope with a discrepancy between the clinical imaging and the 1% labelling suggested by Dr Laverman. ^99m^Tc-DTPA is a hydrophilic compound and, as well stressed by Dr Laverman and colleagues, it is difficult to explain how a hydrophilic radiotracer can pass through the lipid layer and become entrapped in the liposomes. There are two possibilities: (a) our labelling procedure is not efficient and ^99m^Tc-DTPA scanning is the cheapest, still the best, imaging procedure for tumours (which is not true according to ours and the general experience); (b) ^99m^Tc-DTPA labels liposomes without passing through the lipid layer. Caelyx is a pegylated liposome with a water soluble polyethylen-glycol coat. ^99m^Tc-DTPA could be entrapped in this layer and label liposomes without necessitating internalization. The radio-chemical elaboration of labelled-PEG-liposomes on Sephadex performed by Dr Laverman could well lead to ^99m^Tc-DTPA dissociation from the PEG-coat, which explains the poor labelling efficiency found.

Simple labelling procedures of liposomal drugs will become of great value within the following years, when multiple liposomal agents will be available. The technique described by Laverman *et al* (2001) is as simple as ours, while the Indium-oxine labelling allows a better monitoring of the liposome kinetics for at least 3 days after administration. We will certainly incorporate this in current and future projects.
